# Phase I study of the Aurora B kinase inhibitor barasertib (AZD1152) to assess the pharmacokinetics, metabolism and excretion in patients with acute myeloid leukemia

**DOI:** 10.1007/s00280-012-1939-2

**Published:** 2012-08-04

**Authors:** Mike Dennis, Michelle Davies, Stuart Oliver, Roy D’Souza, Laura Pike, Paul Stockman

**Affiliations:** 1 The Christie NHS Foundation Trust, Wilmslow Road, Manchester, M20 4BX UK; 2 AstraZeneca, Alderley Park, Macclesfield, UK

**Keywords:** Barasertib, Acute myeloid leukemia, Pharmacokinetics, Metabolism, Phase I

## Abstract

**Purpose:**

Barasertib (AZD1152) is a pro-drug that rapidly undergoes phosphatase-mediated cleavage in serum to release barasertib-hQPA, a selective Aurora B kinase inhibitor that has shown preliminary activity in clinical studies of patients with acute myeloid leukemia (AML). The pharmacokinetic (PK), metabolic and excretion profiles of barasertib and barasertib-hQPA were characterized in this open-label Phase I study.

**Methods:**

Five patients with poor prognosis AML (newly diagnosed, relapsed or refractory) received barasertib 1,200 mg as a 7-day continuous infusion every 28 days. On Day 2 of Cycle 1 only, patients also received a 2-hour infusion of [^14^C]-barasertib. Blood, urine and feces samples were collected at various time points during Cycle 1. Safety and preliminary efficacy were also assessed.

**Results:**

Barasertib-hQPA was extensively distributed to tissues, with a slow rate of total clearance (CL = 31.4 L/h). Overall, 72–82 % of radioactivity was recovered, with approximately double the amount recovered in feces (mean = 51 %) compared with urine (mean = 27 %). The main metabolism pathways for barasertib were (1) cleavage of the phosphate group to form barasertib-hQPA, followed by oxidation and (2) loss of the fluoroaniline moiety to form barasertib-hQPA desfluoroaniline, followed by oxidation. One of the four patients evaluable for response entered complete remission. No new or unexpected safety findings were observed; the most common adverse events were nausea and stomatitis.

**Conclusions:**

The PK profile of barasertib is similar to previous studies using the same dosing regimen in patients with AML. The majority of barasertib-hQPA clearance occurred via hepatic metabolic routes.

## Introduction

Acute myeloid leukemia (AML) is the most common type of acute leukemia in adults, with a median age of onset of 72 years [1]. The Surveillance Epidemiology and End Results registries analysis found that 86 % of patients aged over 60 years died within 1 year of AML diagnosis [2], attributed in part to their lack of tolerability of intensive chemotherapy regimens, a higher incidence of poor risk cytogenetic abnormalities and a higher incidence of multi-drug resistant gene expression leading to ineffectiveness of chemotherapy [3, 4].

Aurora kinases are a family of serine/threonine protein kinases (Auroras A, B and C) known to play an important role in the regulation of mitosis and chromosomal segregation [5]. Aurora B kinase is involved in the spindle assembly checkpoint component of the mitotic process and is overexpressed in a variety of cancers [6]; several studies have highlighted a role for Aurora B kinase in oncogenic transformation [7, 8]. Increased expression of both Aurora A and Aurora B kinases has been demonstrated in AML cell lines and in primary samples taken from patients with AML [9, 10].

Barasertib is a pro-drug that is rapidly converted into its more active moiety, barasertib-hydroxyquinazoline-pyrazol-aniline (barasertib-hQPA), following parenteral administration in vivo [11]. Barasertib-hQPA is a reversible, selective ATP-competitive inhibitor of Aurora B kinase [11, 12]. Barasertib has demonstrated tumor growth inhibition in solid human cancer tumor xenograft models, such as lung and colorectal cancers [13]. Further, barasertib has been shown to inhibit growth and survival of human AML cells [10, 14]. Clinical studies have indicated that barasertib treatment has an acceptable tolerability profile in patients with newly diagnosed, relapsed, and advanced AML [15, 16]. These studies also included exploratory assessment of the pharmacokinetic (PK) profile of barasertib. In this study, the PK, metabolic and excretion profiles of barasertib and barasertib-hQPA were assessed in patients with newly diagnosed, relapsed or refractory AML (ClinicalTrials.gov identifier: NCT01019161).

## Methods

### Patients

Eligible patients were aged ≥18 years with relapsed or refractory AML for which no standard therapies were anticipated to be effective, or with newly diagnosed AML not considered to be suitable for standard induction and consolidation chemotherapy for medical, social or psychological reasons. All patients were required to have a World Health Organization (WHO) performance status of 0–2; serum creatinine ≤1.5 × upper limit of normal (ULN) or 24-hour creatinine clearance >50 mL/min; serum bilirubin ≤1.5 × ULN (unless considered due to leukemic organ involvement; Gilbert’s or related syndrome allowed); aspartate aminotransferase (AST) or alanine aminotransferase (ALT) ≤2.5 × ULN (also unless considered due to leukemic organ involvement). Exclusion criteria included hemoglobin levels ≤10.0 g/dL; QTc interval ≥470 ms; central nervous system disease; participation in a clinical study in which an investigational product was received within 14 days of the first dose in this study; or if they had received any chemotherapy or radiotherapy within 14 days of start of study treatment.

### Study design

This was an open-label, single-arm, single-center, Phase I study. Patients received barasertib 1,200 mg as a 7-day continuous intravenous infusion every 28 days. On Day 2 of Cycle 1 only, patients also received [^14^C]-barasertib (250 μCi), administered as a 2-hour infusion. Based on preclinical studies in rat and dog, it was expected that an adequate recovery of total radioactivity, and thus comprehensive metabolite profiling, would be achieved following the administration of the specified [^14^C]-barasertib regimen (data on file). On the completion of the [^14^C]-barasertib infusion, administration of non-radiolabeled barasertib resumed for the remainder of the 7-day infusion period. Following Cycle 1, each patient could receive subsequent treatment cycles until disease progression or any other discontinuation criteria were met. For Cycle 1, patients remained as in-patients for up to 9 days following the start of the 7-day barasertib infusion (7 days of dosing plus 2 additional days for observation). For all subsequent cycles, patients were hospitalized for the infusion period only.

The 1,200 mg barasertib dose was the maximum tolerated dose (MTD) previously identified in the Phase I/II dose-escalation study conducted in AML patients [15] and was the therapeutic dose for subsequent Phase II investigation in AML. The amount of radioactivity (250 μCi; 9.25 MBq) administered was considered to be the minimum required to achieve the study objectives. All patients provided written informed consent. The trial was approved by all relevant institutional ethical committees or review bodies and was conducted in accordance with the Declaration of Helsinki, the International Conference on Harmonization/Good Clinical Practice and the AstraZeneca policy on Bioethics [17].

### Study objectives

The primary objective was to assess the PK, metabolism and excretion profiles of barasertib and barasertib-hQPA following a single 7-day intravenous infusion, including a short infusion of [^14^C]-barasertib, to patients with AML. Secondary objectives were to assess the safety and tolerability of barasertib and to evaluate preliminary efficacy by measurement of individual patient response (based on the International Working Group AML clinical response criteria [18]).

### Assessments

Samples of blood, urine and feces were collected prior to study treatment and at various time points during Cycle 1. Blood samples were collected approximately 30 min prior to start of infusion (SOI) on Day 1 (barasertib) and Day 2 ([^14^C]-barasertib), and then every 24 h until Day 10 (216 h) and on Days 14 and 18 (312 and 408 h, respectively). In addition, samples were collected on Day 2: 1 h following the start of the [^14^C]-barasertib infusion ([^14^C]-SOI), 5 min prior to the end of [^14^C]-barasertib infusion ([^14^C]-EOI), and at 15 min, 1, 2, 4 and 8 h following [^14^C]-EOI; and on Day 8: 5 min prior to the end of the barasertib infusion (EOI), and at 15 min, 1, 2, 4, 6 and 10 h following EOI. A urine sample was collected within the 24-hour period prior to barasertib-SOI; feces samples within this 24-hour period were also collected. A urine sample was collected within the 24-hour period following barasertib-SOI. Further, urine and feces samples were collected within 24-hour periods following [^14^C]-SOI: 0–24, 24–48, 48–72, 72–96, 96–120, 120–144, 144–168 and 168–192 h.

Barasertib and barasertib-hQPA concentrations in plasma and urine were measured using liquid chromatography tandem mass spectrometry following solid-phase extraction (plasma) or dilution (urine). Plasma measurements were determined by an independent bioanalytical facility (PRA International—Early Development Services, Assen, The Netherlands). The lower limit of quantification (LLOQ) was 0.25 ng/mL for plasma samples and 1.00 μg/mL for urine samples. Radioactivity in weighed aliquots of plasma, urine, feces, whole blood and dose material was determined by liquid scintillation counting (LSC). Radioactivity concentrations in plasma and whole blood, and percentage dose excreted in urine and feces samples were calculated using LabLogic Debra 5TM (version 5.4.10.51). For metabolite profiling, plasma samples that were collected 5 min prior to [^14^C]-EOI for each patient were prepared using 5 % formic acid in acetonitrile; extraction efficiencies were all >91 %. Extracts were concentrated to dryness and reconstituted in water/acetonitrile (95:5 v/v). Pooled urine samples were freeze-dried and then reconstituted in methanol (duplicate extracts); extraction efficiencies were all >94 %. The two extracts were combined, concentrated to dryness and reconstituted in water/acetonitrile (95:5 v/v). Pooled feces samples were extracted with hexane followed by 3 × 5 % formic acid in acetonitrile; extraction efficiencies ranged from 86 to 98 %. The first two 5 % formic acid/acetonitrile extracts were combined, concentrated to a small volume and reconstituted in water/acetonitrile (95:5 v/v). Metabolite profiling was determined using high-performance liquid chromatography–radiometric (HPLC-RAD) methodology, with metabolites identified by HPLC coupled with mass spectrometry (HPLC-MSn).

Efficacy was assessed according to the International Working Group for AML trials criteria [18]. Adverse events (AEs) were graded according to the National Cancer Institute Common Terminology Criteria for Adverse Events (CTCAE), version 3.0 [19].

### Statistical analysis

No formal statistical analyses were planned; all objectives were assessed descriptively. Previous experience with mass balance studies found that a sample size of six patients was sufficient to adequately characterize the rates and routes of excretion of [^14^C]-labeled compounds [20, 21]. All patients who received at least one dose of barasertib and for whom post-dose data were available were included in the assessment of safety and efficacy. Patients were included in the PK analysis if they had sufficient blood sampling to determine key endpoints.

## Results

### Patients

Between November 2009 and June 2010, six patients were enrolled in this study. One patient died before receiving any study treatment; the remaining five completed Cycle 1 and were therefore included in the PK and safety analysis sets. Three of these five patients completed at least one further treatment cycle. One patient started Cycle 2 on Day 36 after a 7-day delay due to logistical reasons; this patient also started Cycle 3 on Day 78 but discontinued treatment on the same day due to worsening of the disease. The second patient also started Cycle 2 on Day 36 after a 7-day delay in order to allow for hematological recovery and subsequently received Cycle 3, starting on Day 64. The third patient started Cycle 2 on Day 57 for logistical reasons. The baseline demographic and disease characteristics were representative of the intended patient population for this study (Table 1). At the time of data cutoff (August 20, 2010), and for those patients who had received barasertib, one patient was still on treatment, two patients had progressed, one patient had withdrawn due to lack of response, and one patient had died due to an AE.

**Table 1:** Patient demographics and baseline characteristics

	Barasertib 1,200 mg n = 6
Median age (range), years	63 (34–74)
Male/female, *n*	3/3
Median body weight (range), kg^a^	66 (55–85)
Race, *n*	
Caucasian	4
Asian	2
WHO performance status, *n*	
0	1
1	4
Missing	1
AML type, *n*	
De novo	4
Secondary to MDS	2
AML status, *n*	
Newly diagnosed	2
First relapse	1
Second relapse	3

*WHO,* World Health Organization; *MDS,* myelodysplastic syndrome

^a^Data missing for one patient that died prior to receiving study treatment

### Pharmacokinetic parameters

**Fig. 1:**
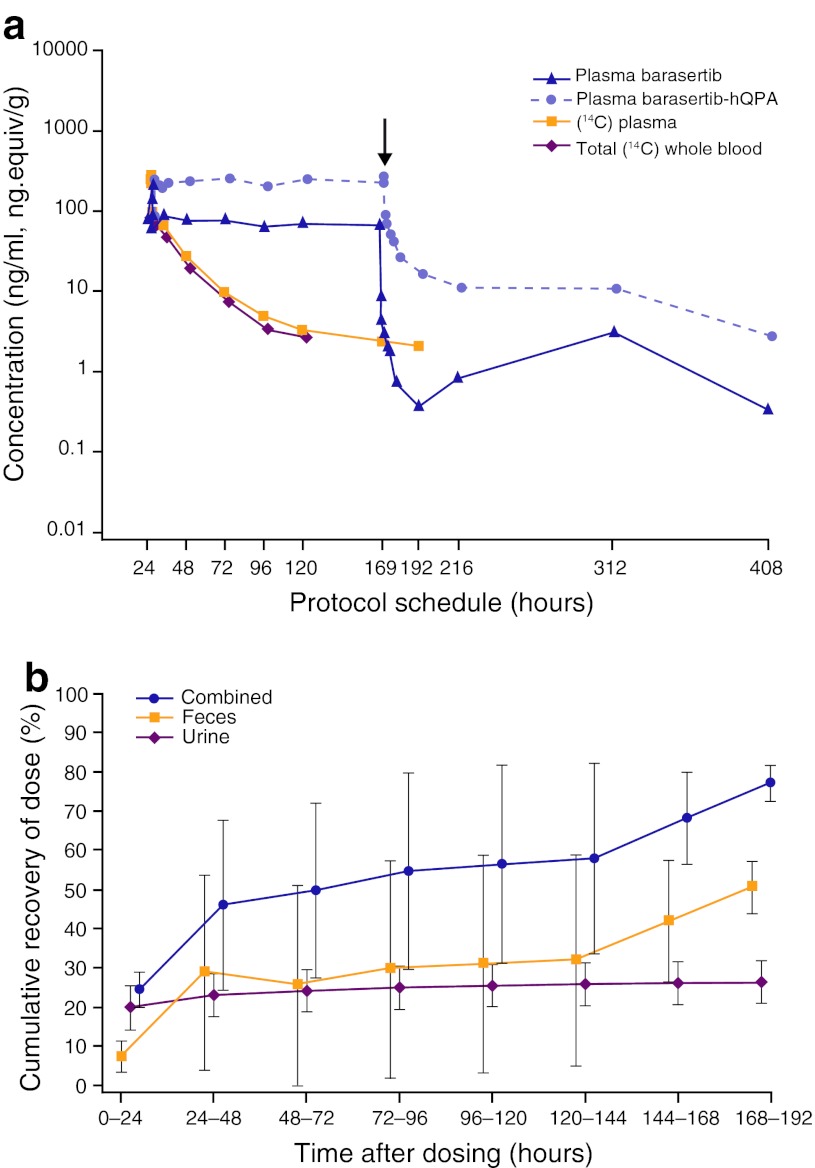
Barasertib and barasertib-hQPA recoverability **a** Geometric mean plasma concentrations of barasertib and barasertib-hQPA, and total radioactivity [^14^C] in plasma and whole blood, versus time. *Arrowhead* indicates start of barasertib infusion; *arrow* indicates end of barasertib infusion. **b** Cumulative mean (± standard deviation) percentage radioactivity dose recovered in urine, feces and combined. The assessment time points are from the start of ^14^C-SOI

Maximal plasma concentrations of barasertib and barasertib-hQPA were achieved by the first scheduled sample, taken 24 h from the SOI (Fig. 1a). During the infusion period, the geometric mean plasma concentration of barasertib-hQPA was approximately threefold higher than that of barasertib (Table 2); this is consistent with previous studies [15, 16]. Following the EOI, plasma concentrations of barasertib fell rapidly, reaching the LLOQ by 6 h after EOI; it was therefore not possible to determine the terminal elimination half-life (t_½_), total clearance (CL) or volume of distribution (V) of barasertib. In contrast, barasertib-hQPA plasma levels declined in a triphasic manner, with a rapid initial phase (with plasma concentrations decreased to one-third of the plasma steady-state concentration within 2 h post-EOI) followed by a slower decline thereafter. Low concentrations (~4 ng/mL) of barasertib-hQPA were still detectable at the final sampling point of 408 h (Day 18; Fig. 1a); the terminal elimination phase had a mean t_½_ of 66.3 h (Table 2). Barasertib-hQPA was extensively distributed to the tissues, with a relatively slow rate of total clearance (Table 2). For four patients, maximum plasma radioactivity concentration was achieved 5 min prior to [^14^C]-EOI; for the remaining patient, this was achieved 1 h prior to [^14^C]-EOI. The concentrations of radioactivity in whole blood were lower than in plasma (with a ratio approximating 0.7) at all time points examined (Fig. 1a). Urine concentrations of barasertib were below LLOQ at all time points; during the infusion period, the geometric mean urine concentrations of barasertib-hQPA were approximately 4–5 μg/mL, declining rapidly following EOI. The renal clearance values for barasertib-hQPA represent approximately 10 % of the total clearance of barasertib-hQPA from plasma (Table 2).

**Table 2:** Pharmacokinetics of barasertib and barasertib-hQPA in plasma and urine

Sample (*n*)	Parameter^a^	Barasertib	Barasertib-hQPA
Plasma (4)^b^	AUC, ng.h/mL^c^	NC	40,400 (0.4)
	AUC_(0–t)_, ng.h/mL^c^	13,840 (0.4)	40,080 (0.4)
	AUC_(0–192 h)_, ng.h/mL^c^	NC	38,230 (0.4)
	C_SS_, ng/mL^c^	85.6 (0.5)	226.8 (0.4)
	CL, L/h	NC	31.4 (11.3)
	t_½_, h	NC	66.3 (36.1)
	λ_Z_, h–1	NC	0.02 (0.02)
	V_SS_, L	NC	669.1 (344.9)
	V_Z_, L	NC	2805 (1708)
Urine (5)	Ae, mg	NC	96.9 (47.2)
	CL_R_, L/h	NC	2.85 (1.6)
	fe %	NC	8.1 (3.9)

*AUC,* area under plasma concentration–time curve; *C*_*SS*_, steady-state drug concentration; *CL,* total clearance; *t*_*½*_, terminal half-life; *λ*_*Z*_, slowest disposition rate constant; *V*_*SS*_, volume of distribution (apparent) at steady state; *V*_*Z*_, volume of distribution (apparent) during terminal (λ_Z_) phase; *Ae,* cumulative amount of unchanged drug excreted; *CL*_*R*_, renal clearance of drug from plasma; *fe,* fraction of drug excreted into urine; *NC,* not calculable

^a^All values are arithmetic mean (standard deviation) except where indicated. ^b^ Due to sampling issues, plasma concentration data were excluded for one patient. ^c^ Values indicate geometric mean (coefficient of variation)

### Recovery of total radioactivity

The target dose of [^14^C]-barasertib was 250 μCi; the actual dose received ranged from 231 to 268 μCi. Overall, 72–82 % of total radioactivity was recovered, with approximately double the amount recovered in feces (mean ± SD, 51 ± 6.6 %) compared with urine (mean ± SD, 27 ± 5.5 %; Fig. 1B). There was large inter-patient variability in the rate of recovery of radioactivity in feces: the majority (>40 %) of radioactivity was recovered by 120 h in three patients, while in the remaining two patients, the majority (>44 %) was recovered during the last two time points (144–192 h) and it was evident that radioactivity was still being eliminated in feces after the end of the sample collection period. In contrast, the excretion of radioactivity in urine occurred predominantly within 72 h and was almost complete by 96 h from the [^14^C]-SOI.

### Metabolite profiling

**Fig. 2:**
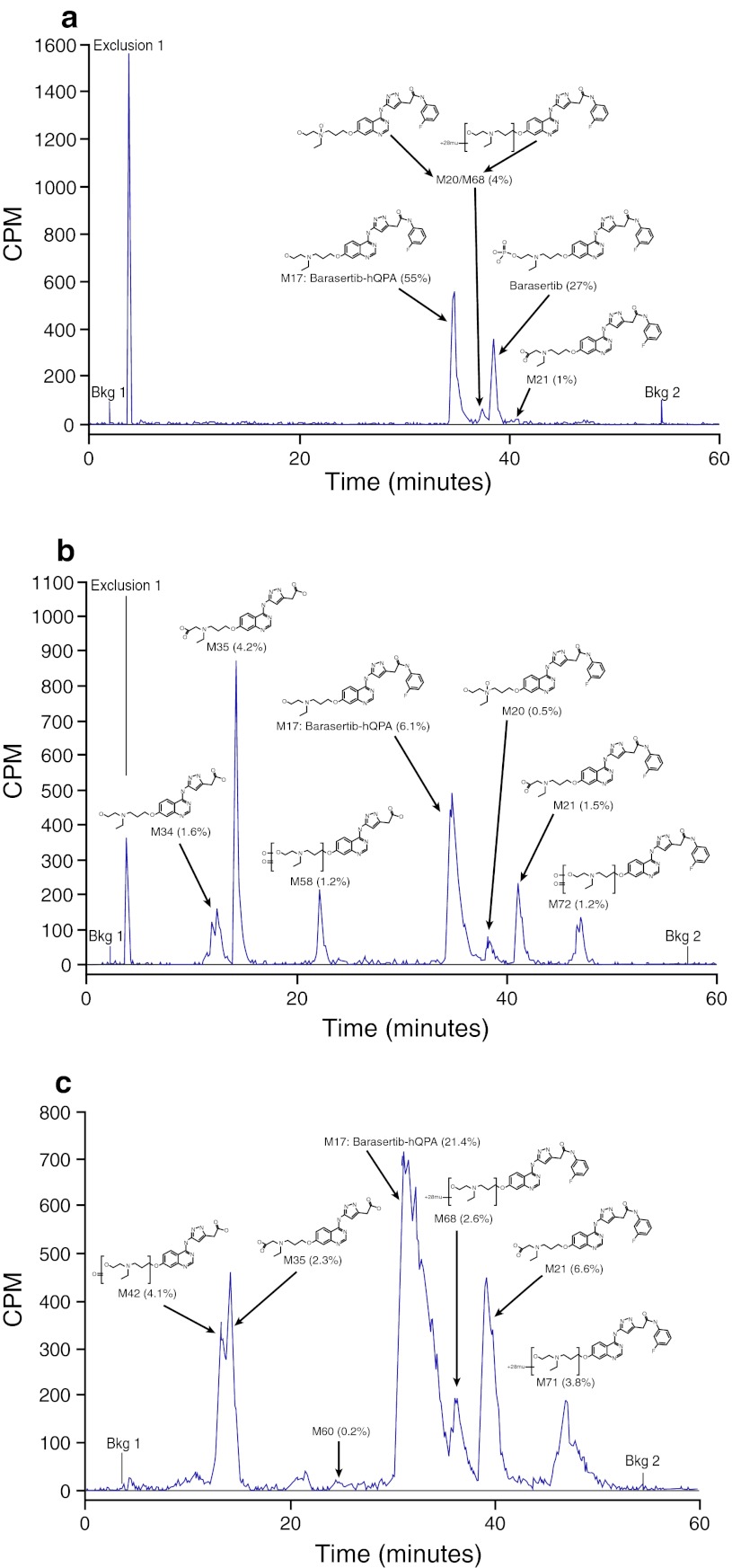
Representative HPLC-RAD chromatographs for analyses of plasma, urine and feces samples. **a** Plasma sample collected 5 min prior to the end of the [^14^C]-barasertib infusion; **b** Urine sample collected 0–24 h from start of the [^14^C]-barasertib infusion; **c** Pooled feces sample collected 144–192 h from start of the [^14^C]-barasertib infusion.* CPM* counts per minute

Representative chromatographs for HPLC-RAD analyses of plasma, urine and feces samples are presented in Fig. 2. In plasma, the main metabolite was barasertib-hQPA; a large proportion of unmetabolized barasertib was also detected in plasma samples. Overall, the main excreta metabolites identified were the following: barasertib-hQPA (range, 13.2–33.7 %); barasertib-hQPA N-acetic acid (range, 7.8–10.5 %); barasertib-hQPA desfluoroaniline N-acetic acid (range, 5.1–9.5 %); N-formyl or ethoxy barasertib-hQPA (range, 3.5–9.2 %); and barasertib-hQPA desfluoroaniline (range, 1.6–4.5 %). Unlike plasma, desfluoroaniline metabolites made up a significantly larger proportion of the metabolites in excreta (~15 % compared with ~2 % in plasma) (Table 3). No glutathione or epoxide metabolites were detected in any samples. The main pathways identified in human metabolism of barasertib were (1) cleavage of the phosphate group to form barasertib-hQPA, followed by oxidation; and (2) loss of the fluoroaniline moiety to form barasertib-hQPA desfluoroaniline, followed by oxidation.

**Table 3:** [^14^C]-barasertib and metabolite peaks identified in plasma and excreta extracts; components >1 % of chromatogram radioactivity or dose are listed

	HPLC metabolite	Retention time, range (min)	Protonated molecular ion [M + H]^+^	Retrieved radioactivity (%), range^a^
Plasma^b,c^	Barasertib-hQPA	34.33–35.20	508	48.27–56.61
	Barasertib	37.97–38.57	588	17.26–26.53
	Barasertib-hQPA N-acetic acid	40.43–40.77	522	1.42–5.29
	N-formyl or ethoxy barasertib-hQPA	37.00–37.87	536	4.24–4.42
	Barasertib-hQPA desfluoroaniline N-acetic acid	14.33–14.71	429	1.33–2.21^d^
	Barasertib-hQPA desfluoroaniline	11.30–11.70	415	2.05^e^
Excreta	Barasertib-hQPA	31.00–35.50	508	13.15–33.70
	Barasertib-hQPA N-acetic acid	39.10–41.47	522	7.83–10.52
	Barasertib-hQPA desfluoroaniline N-acetic acid	13.70–18.07	429	5.07–9.45
	N-formyl or ethoxy barasertib-hQPA	46.13–47.10	536	3.47–9.24
	Barasertib-hQPA desfluoroaniline	11.57–18.07	415	1.60–5.48
	Ketone of barasertib-hQPA desfluoroaniline	10.67–13.13	429	1.73, 4.88^e^
	Ketone of barasertib-hQPA desfluoroaniline	4.30–4.90	429	0.88–4.19
	N-formyl or ethoxy barasertib-hQPA	36.00–39.07	536	0.96–3.86
	Barasertib-hQPA N-oxide	37.63–38.60	524	0.21–2.96
	Ketone of barasertib-hQPA desfluoroaniline	13.70–14.20	429	0.34, 2.37^f^
	Di-oxygenated dehydrogenated barasertib-hQPA	46.47–46.97	538	0.18–1.20
	Di-oxygenated dehydrogenated barasertib-hQPA desfluoroaniline	22.00–23.19	445	0.17–1.19
	Ketone of barasertib-hQPA or N-desethyl N-acetyl metabolite	43.00–44.70	522	5.65^g^

^a^Data presented for plasma are expressed as % of chromatogram radioactivity; data presented for excreta are presented as % of administered dose

^b^Measurements from one patient are omitted due to sampling error

^c^Two unidentifiable metabolites were detected in two patients

^d^Confirmed by mass spectrometry only in one patient

^e^Confirmed by mass spectrometry only in three patients

^f^Detectable in two patients only

^g^Detectable in one patient only

### Safety

There were no dose interruptions or dose reductions during the study. The most common AEs (any grade; any cause) were nausea and stomatitis (n = 4 each); stomatitis and febrile neutropenia were the only grade ≥3 AEs reported in more than one patient (Table 4). Grade ≥3 AEs were experienced by three patients. One patient experienced febrile neutropenia and stomatitis (both grade 3); a second patient reported febrile neutropenia, stomatitis, anemia, abdominal distension, gingival bleeding, neutropenic sepsis, hypocalcemia, hypomagnesemia and lethargy (all grade 3). The third patient experienced febrile neutropenia and thrombocytopenia (both grade 4); this patient subsequently experienced grade 5 multi-organ failure, which was the only death during the study and was considered unrelated to study treatment (this patient had marrow failure with neutropenia as a consequence of AML; this became complicated by septicemia). Abnormalities in some hematological, clinical chemistry and urinalysis results were observed, which were consistent with the patients’ underlying disease. There were no clinically relevant changes in vital signs or ECG measurements.

**Table 4:** Adverse events (any cause) occurring in ≥2 patients

	Number of patients		
	Barasertib 1,200 mg n = 5		
	All grades	Grade 3	Grade 4
Nausea	4	–	–
Stomatitis	4	2	–
Alopecia	3	–	–
Febrile neutropenia	3	2	1
Hypocalcemia	3	1	–
Vomiting	3	–	–
Constipation	2	–	–
Decreased appetite	2	–	–
Diarrhea	2	–	–
Fatigue	2	–	–
Headache	2	–	–
Hypomagnesemia	2	1	–
Oral candidiasis	2	–	–
Peripheral edema	2	–	–

### Efficacy

Four patients were evaluable for disease response. One patient achieved a complete response (commenced Cycle 3, Day 92; duration ~6 months; a 73-year-old female with a complex abnormal karyotype who had previously failed hypomethylating therapy); the cytogenetic profile of this patient at screening was “adverse complex.” No response was observed in the remaining three patients.

## Discussion

This small Phase I study defined the PK, metabolic and excretion profiles of barasertib and barasertib-hQPA in patients with newly diagnosed, relapsed or refractory AML. We do not consider that the interpretation of our results is substantially affected by the smaller than prespecified study population (n = 5). The PK profiles of barasertib and barasertib-hQPA determined here are in agreement with those previously reported for patients with advanced AML [15, 16], and similar findings have also been reported in patients with solid tumors following barasertib administration by various infusion schedules [22]. Barasertib is rapidly absorbed, and conversion to barasertib-hQPA leads to an approximate threefold higher mean plasma concentration of barasertib-hQPA compared with barasertib. As observed previously, the fourfold difference between the volumes of distribution (apparent) during steady state versus the terminal phase indicates that the majority of barasertib-hQPA is eliminated before the distribution equilibrium in the tissue is achieved. Thus, the time to a steady-state concentration of barasertib-hQPA is governed by distribution kinetics rather than its much longer t_½_ (arithmetic mean, 66.3 h). Further, the ratio of radioactivity in plasma to whole blood (approximately 0.7) indicated that barasertib is associated with a greater degree with the plasma fraction compared with the cellular components of the blood. Data from a previous in vitro study showed that between 89.8 and 97.2 % of barasertib was bound to human plasma proteins (data on file).

From the PK analysis, the renal clearance values for barasertib-hQPA (4–15 % of the total clearance of barasertib-hQPA from plasma) suggested that the majority of clearance of barasertib-hQPA occurred via non-renal, hepatic metabolic routes. From our radiolabeled studies, we identified that the major route of elimination was via feces, although the recovery rate showed high inter-patient variability.

This study is the first to characterize the metabolic pathway of barasertib in humans. Metabolites were observed in this study that had not been previously identified in rat or dog plasma and excreta (data on file), suggesting that these species may not provide good models for the metabolic fate of barasertib in man. Barasertib is metabolized by a number of pathways in humans. The predominant pathway is the conversion to barasertib-hQPA, which is subsequently followed by oxidation. Another major metabolic pathway leads to the loss of the fluoroaniline moiety to form a desfluoroaniline metabolite, also followed by oxidation. Cleavage of the nitrogen bond between the quinazoline and pyrazole ring systems followed by oxidation and/or conjugation was found to be a minor pathway. Another minor pathway was cleavage of the ethyl group on the alkyl side chain of barasertib-hQPA. Fluoroaniline metabolites detected were mainly conjugates (both glucuronides and sulfates), and no glutathione or epoxide metabolites were detected in any samples. In plasma, the major components were barasertib and barasertib-hQPA, indicating that the majority of metabolism occurs once the drug has been distributed to the tissues.

In our study, no new or unexpected safety findings were reported with barasertib. The most common AEs were nausea and stomatitis; stomatitis and febrile neutropenia were the only grade ≥3 AEs reported in more than one patient. The AEs encountered were as expected for this patient population and are consistent with those reported in previous studies of barasertib in AML [15, 16].

One complete remission was reported in this small patient population, suggesting that barasertib monotherapy may have potential as a treatment strategy in this challenging setting. Indeed, in Phase I studies in both Japanese and Western patients with AML, treatment with barasertib was associated with response rates of 19–25 % [15, 16]. The preliminary efficacy observed in this patient population warrants further investigation. Studies are also assessing the combination of barasertib and low-dose cytosine arabinoside in patients aged ≥60 years with AML; in one Phase I, safety and tolerability study, the combination was found to have an acceptable tolerability profile with a preliminary signal for efficacy [23].

In conclusion, barasertib and barasertib-hQPA exhibited similar PK profiles to those previously observed in patients with solid tumors and AML. We have characterized the metabolic profile of barasertib and have shown that barasertib metabolites are eliminated predominantly via the feces. The safety profile of barasertib was as expected, and the drug was tolerated in this population. Preliminary efficacy findings indicate potential for benefit in this patient population, which warrants further investigation.
